# Dr. Emil Zuckerkandi's

**Published:** 1910-06-04

**Authors:** 


Dr. Emil Zuckerkandl's world-wide reputation as a teacher of anatomy makes the news of his death, at the age of 61, a loss to scientists everywhere. He died at Vienna, where he held the post of professor of anatomy at the University. It will be remembered that, among his many writings, the subject to which he devoted most study was the physiology of the glands.

